# Finnish *Campylobacter jejuni* Strains of Multilocus Sequence Type ST-22 Complex Have Two Lineages with Different Characteristics

**DOI:** 10.1371/journal.pone.0026880

**Published:** 2011-10-24

**Authors:** Joana Revez, Mirko Rossi, Patrik Ellström, Caroline de Haan, Hilpi Rautelin, Marja-Liisa Hänninen

**Affiliations:** 1 Department of Food Hygiene and Environmental Health, Faculty of Veterinary Medicine, University of Helsinki, Helsinki, Finland; 2 Department of Medical Sciences, University of Uppsala, Uppsala, Sweden; 3 Department of Bacteriology and Immunology, Haartman Institute, University of Helsinki and HUSLAB, Helsinki University Central Hospital Laboratory, Helsinki, Finland; Charité-University Medicine Berlin, Germany

## Abstract

**Background:**

*Campylobacter jejuni* is the major cause of human bacterial gastroenteritis worldwide, and in a minority of cases, post-infectious complications may occur. ST-22 complex (usually Penner serotype 19) strains have been overrepresented among patients with postinfectious complications of campylobacteriosis. We here present a characterization of a collection of 27 Finnish *C. jejuni* strains of ST-22 complex, from humans (22 strains) and animal sources (five strains), with the aim of contributing to our knowledge of the pathogenesis of *C. jejuni* infections.

**Methodology/Principal Findings:**

All strains were analyzed by pulsed-field gel electrophoresis (PFGE) genotyping, lipo-oligosaccharide (LOS) locus class, Y-glutamyl transpeptidase (GGT) activity, *in vitro* biofilm formation ability, invasion and adhesion in HeLa cells and induction of IL-8 production. ST-22 complex contained five STs (ST-22; ST-1947; ST-1966; ST-3892; ST-3996) which were homogeneous in having sialylated LOS class A_1_ but on the other hand were distinguished into two major lineages according to the major STs (ST-22 and ST-1947) by different PFGE genotypes and certain other characteristics. All ST-22 strains had similar *Sma*I PFGE profiles, were GGT positive, and formed biofilms, except one strain, while ST-1947 strains were all GGT negative, did not form biofilm, had significantly higher motility than ST-22 (*p<*0.05) and had their *Sma*I PFGE profile. Invasion and adhesion as well as induction of IL-8 production on HeLa cells were strain-dependent characteristics.

**Conclusions/Significance:**

ST-22 complex strains, reveal potential for molecular mimicry in host interactions upon infection as they all express sialylated LOS class A_1_. The two major STs, ST-22 and ST-1947 formed two homogeneous lineages, which differed from each other both phenotypically and genetically, suggesting that the strains may have evolved separately, perhaps by interacting with different spectra of hosts. Further studies are needed in order to understand if these two lineages are associated with different disease outcomes.

## Introduction

*Campylobacter jejuni* is the most common bacterial cause of human gastroenteritis throughout the industrialized world. In 2010 the reported incidence of campylobacteriosis in Finland was 74 cases per 100,000 inhabitants, according to the Finnish National Infectious Diseases Registry (http://www3.ktl.fi/stat/). Despite the importance of *C. jejuni* as enteric pathogen and the progress in recent years in comprehending the complicated and multifactorial pathogenesis, critical gaps remain about the mechanisms causing the disease [1], [2]. Indeed there is a gap in understanding the combination of phenotypic and genotypic characteristics that have relevance for pathogenicity and also in survival both in the environment and in the food chain [2], [3]. Not all *C. jejuni* strains appear to have the same virulence, survival and host adaptation potential as well as other characteristics. For instance, the ability to invade cultured cells as well as certain metabolic activities appear to be strain dependent [1]. Genetic studies have shown that *C. jejuni* isolates which appear highly related based on their Multi Locus Sequence Types (ST) can have remarkable differences in their genomic content [2]. The high population diversity has been identified already earlier using several other techniques, such as serotyping or pulsed-field gel electrophoresis (PFGE) genotyping [4]. Multi Locus Sequence Typing (MLST) and the establishment of the international accessible MLST database (PubMLST- http://pubmlst.org/campylobacter) [5], has offered an objective tool to analyse the diversity and global distribution of different ST complexes and types of *C. jejuni*. Currently, a total of 5096 STs have been detected among 11168 isolates published in the database, the most frequent clonal complexes being ST-21 complex (15.1%), ST-45 complex (7.5%) and ST-257 complex (4.1%). On the other hand, these frequencies do not reflect the actual prevalence in different countries, since limited numbers of isolates representing various STs are submitted to the database. Genetic diversity within a clonal complex can be extensive due to recombination as found among ST-45 complex [2]. On the other hand, certain ST-complexes, such as ST-22 (Penner serotype 19) and ST-403 (Penner serotype 23), have shown that they may represent clonal lineages among *C. jejuni* populations [6]–[8].

Most human infections seem to be sporadic and the gastrointestinal symptoms are usually self-limiting. However, a minority of patients may develop extraintestinal complications such as reactive arthritis (ReA), Guillain-Barré (GBS) and Miller-Fisher (MFS) syndromes [6], [9]. Although there are no reports regarding the occurrence of GBS or MFS related to campylobacteriosis in Finland, ReA has been quite frequently detected [9]–[11]. These postinfectious complications are suggested to be a result of an autoimmune response induced by ganglioside-like lipooligosaccharides (LOS) expressed by *C. jejuni* [12]–[14]. In addition to molecular mimicry, it has been shown that sialylated LOS are also implicated in the interaction with host cells and in the modulation of the immune response [15]–[17], representing an important virulence determinant. Interestingly, even if ST-22 complex constitutes only 1.3% of all the submitted isolates in the PubMLST database, it accounts for 31% of all *C. jejuni* associated GBS cases [7], [8], [18], [19]. We recently characterized MLST types among 454 human isolates and found that the ST-22 complex was rather common in two seasonal peaks with 13% in 1996 (predominantly ST-22) and 11% in 2006 (predominantly ST-1947) of all isolates studied [20].

In the present study, we characterized phenotypes (lipo-oligosaccharide locus class, Y-glutamyl transpeptidase (GGT) activity, *in vitro* biofilm formation ability, invasion and adhesion capability and induction of IL-8 production in HeLa cells) and genotypes (pulsed-field gel electrophoresis) of 27 Finnish *C. jejuni* strains belonging to the ST-22 complex in order to identify bacterial properties which could contribute to what is known of the pathogenesis of *C. jejuni* infections. All the strains had sialylated LOS class A_1_, revealing a potential for molecular mimicry in host interactions upon infection processes. However, the strains formed two distinct lineages with great homogeneity within each lineage, but that differed from each other both phenotypically and genotypically. These data suggest that these lineages have evolved separately, perhaps by interacting with different spectra of hosts.

## Results

### Genotyping of the strains

**Figure 1:**
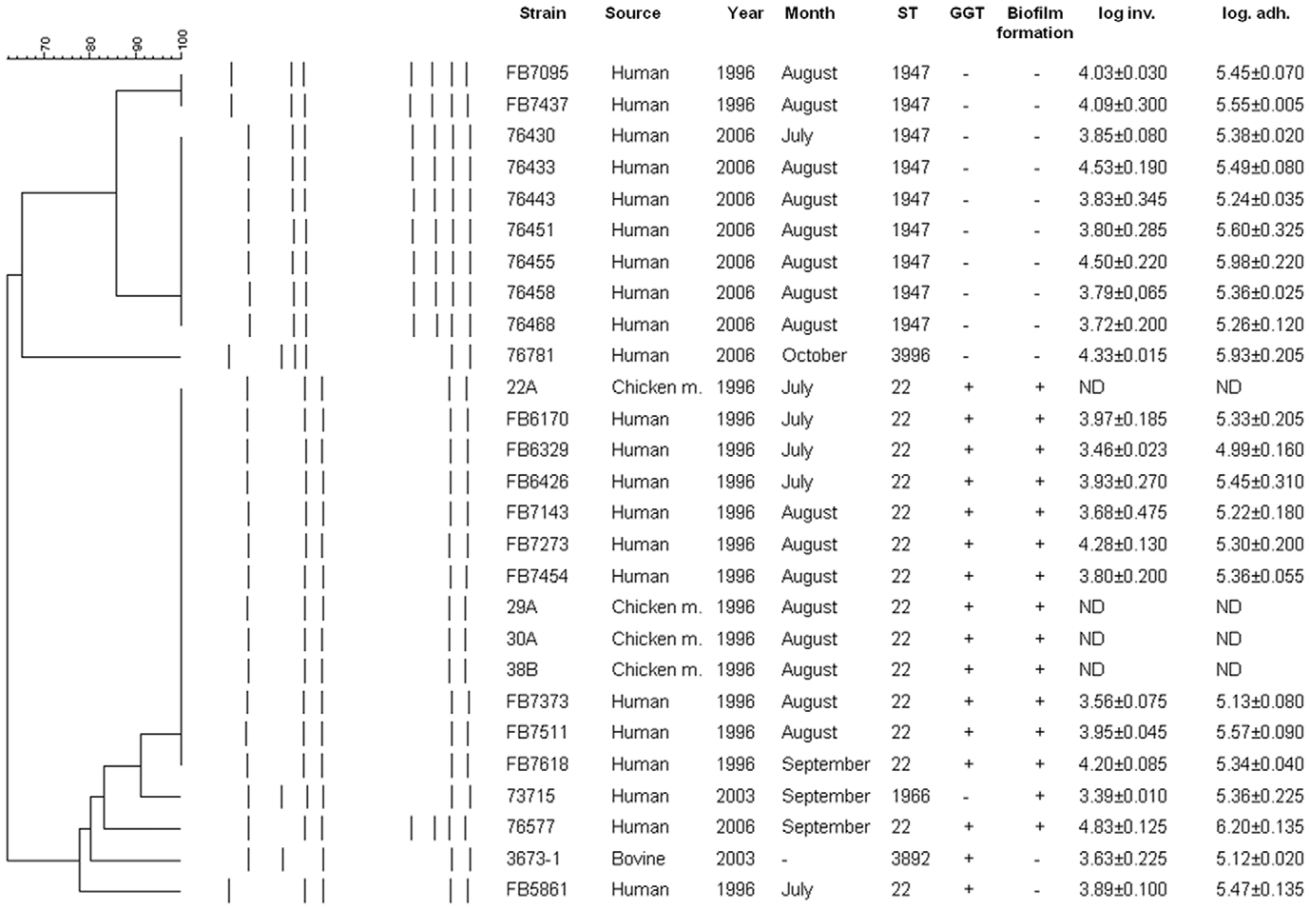
Phenotypic and genotypic characteristics of the 27 strains of *Campylobacter jejuni* of the ST-22 complex. Distribution of *Sma*I digested PFGE patterns, GGT production, biofilm formation and invasion and adhesion on HeLa cells (expressed in log CFU/ml) of the strains are shown. ND: Not Done.

PFGE analysis with *Sma*I subdivided the strains mostly according to their ST types (Figure 1) clustering ST-1947 and ST-22 strains into different branches of the tree. PFGE genotypes of the three strains with unique ST types (human strains 73715 and 76781 and bovine strain 3673-1), were all different. The unique STs clustered either within the ST-1947 branch (ST-3996) or along with the ST-22 strains (ST-3892 and ST-1966). Strain FB5861 had a PFGE genotype which differed by one fragment from the major genotype of this group. PFGE patterns of the four chicken meat *C. jejuni* isolates from 1996 (ST-22) were identical to those of human ST-22 strains from 1996.

### LOS biosynthesis class and SDS-PAGE

All the strains belonged to LOS locus class A_1_ according to the PCR screening analysis and their LOS profiles in SDS-PAGE were all identical. In addition all strains contained sialic acid, which was confirmed by the treatment with neuraminidase (data not shown).

### *In vitro* phenotypic studies

**Figure 2:**
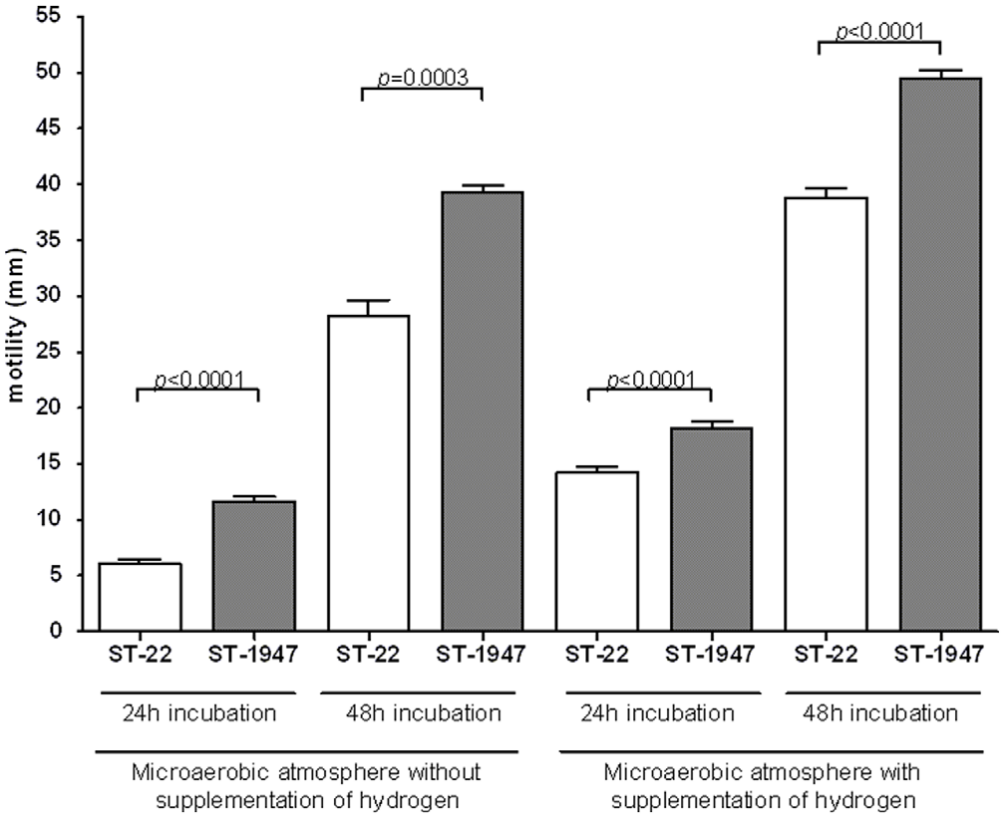
Motility of ST-22 and ST-1947 within the ST-22 complex. The motility of a total 27 *C. jejuni* strains was evaluated on soft agar (Muller-Hinton supplemented with 0.4% of agar) at 37°C after 24h and 48h, incubated microaerobically with or without hydrogen enrichment. White bars: ST-22 strains, Grey bars: ST-1947 strains. Strains exhibited higher motility when incubated microaerobically with hydrogen and the ST-22 strains, were significantly less motile (*p*<0.05) than the ST-1947 strains in all conditions tested. All *p* values derived from unpaired two tailed t tests. Data are presented as mean ± SEM.

Results for biofilm formation ability and GGT production are presented in Figure 1. In general, all, except one of ST-22 strains, were able to form biofilms, whereas none of ST-1947 strains had the ability to form biofilms on the borosilicate glass tubes. Strains 76781 (ST-3996) and 3673-1 (ST-3892) were not able to form biofilm whereas strain 73715 (ST-1966) did form a biofilm. The final OD_600_ for the planktonic phase for all strains was 0.10±0.01 (mean±SD). All ST-22 strains had GGT activity while ST-1947 strains did not. The strains with unique STs were either GGT positive (ST-3892) or negative (ST-3996 and ST-1966). All strains were motile, although the average diameter of the zone of motility was dependent on incubation conditions (with or without supplementation of hydrogen) as well as on STs (Table S1). Motility of the strains with ST-22 and ST-1947 on soft agar is presented in Figure 2. In general, strains exhibited higher motility when incubated microaerobically with hydrogen. The ST-22 strains, except for the FB5861 strain, were significantly less motile (*p*<0.05) than the ST-1947 strains both in different incubation atmospheres as well as incubation period. However, when strains were incubated microaerobically with hydrogen, differences among STs were less accented, but nevertheless statistically significant. Moreover, in the soft agar supplemented with 20 mM of Gln and GSH, the motility of all strains increased but the difference between the ST-22 and ST-1947 strains remained statistically significant (*p<*0.05). In opposition, in the media supplemented with 20 mM of Glu, no changes were observed comparing to the Mueller Hinton soft agar (data not shown). No significant associations were found between these characteristics and the ability to adhere, invade and/or stimulate IL-8 production in HeLa cells (*p>*0.05).

### Adhesion, Invasion and IL-8 production

Overall, all the bacterial strains adhered to HeLa cells in a range of 0.214–5.374% of the inoculated bacteria and were internalized in HeLa cells at a range 0.005–0.229% of the inoculated bacteria. ST-22 strains adhered and invaded HeLa cells 5.40±0.07 log CFU/mL and 3.96±0.09 log CFU/mL, respectively. The adhesion average of ST-1947 strains was 5.47±0.06 log CFU/mL while invasion was 4.01±0.09 log CFU/mL. Strains with an unique ST adhered to HeLa cells 5.47±0.170 log CFU/mL and invaded 3.78±0.187 log CFU/mL (Figure 1). The correlation between STs and respective adhesion and invasion in HeLa cells was not statistically significant (*p>*0.05).

**Figure 3:**
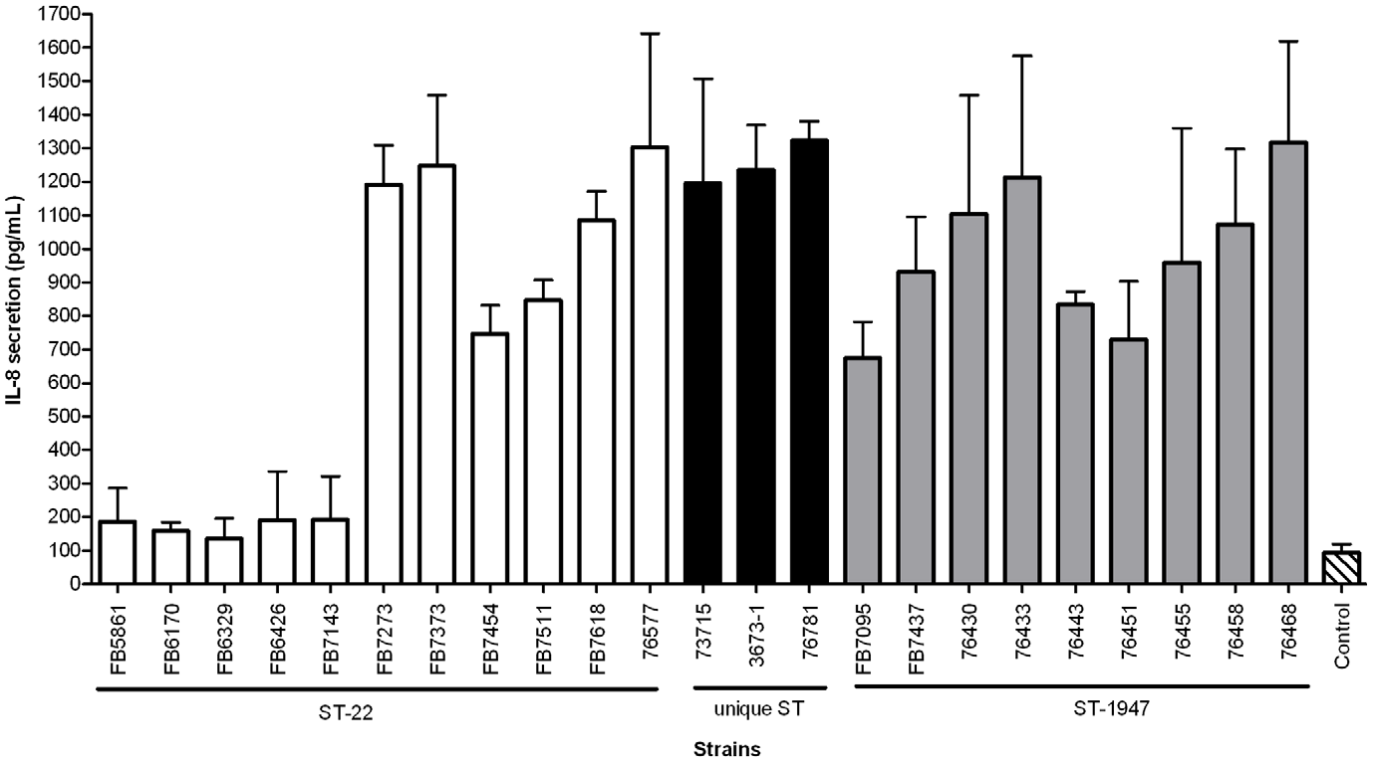
IL-8 secretion induced from *Campylobacter jejuni*-HeLa cells co-culture after 24h of incubation. White bars represent ST-22 strains, black bars represent unique ST (1966, 3892 and 3996, respectively) and gray bars represent strains with ST-1947. The control (or basal level of IL-8 secretion by HeLa cells) is represented by the striped bar. The difference between ST groups was not significant (*p>*0.05). Five strains belonging to the ST-22 (FB5861, FB6170, FB6329, FB6426 and FB7143) promoted significantly lower IL-8 secretion on HeLa cells than the other strains with ST-22, ST-1947 and unique STs (*p<*0.001). The four chicken strains were not done. Data are presented as mean ± SEM.

*C. jejuni*-infected HeLa cells showed elevated IL-8 secretion (Figure 3). The average of IL-8 secretion by HeLa cells (expressed in pg/mL±SD) was 662.2±432.9 for ST-22 strains, 981.8±315.5 for ST-1947 strains and 1251±62.7 for strains with unique STs. The basal level of IL-8 secretion of HeLa cells averaged 93.3±26.53 pg/mL. The difference between ST groups was not significant (*p>*0.05). However, five strains belonging to the ST-22 (FB5861, FB6170, FB6329, FB6426 and FB7143) promoted significantly lower IL-8 secretion on HeLa cells than the other strains with ST-22, ST-1947 and unique STs (*p<*0.001).

## Discussion

In the current study we analysed a total of 27 *C. jejuni* strains, either from human patients or chicken meat or bovine faecal matter, encompassing to our knowledge the largest collection of strains of the ST-22 complex so far described. ST-22 complex have been detected among *C. jejuni* strains of human and animal origin in several countries, although with a low frequency compared with many other clonal complexes [7], [19]–[23]. However, the ST-22 complex has been a target of interest, since its association with severe postinfectious complications, such as GBS [7], [24]. In our study, all the strains of the ST-22 complex had sialylated LOS locus class A_1_ and thus showed potential for molecular mimicry in host interaction, but otherwise the two major STs in the complex differed from each other in many phenotypical and genotypical features.

The 22 human ST-22 complex *C. jejuni* strains were isolated from patients who had acquired the disease from domestic sources between June and October either in 1996 or 2006 and were subdivided by both MLST and PFGE typing into two major groups. Interestingly, ST-22 complex strains were detected from patients only in 1996 and 2006, except for one human strain from 2003 during our ten-year follow up study of human strains from the same region [20], [25], [26]. It is remarkable to note that in 1996 the type was mainly ST-22 (10 out of 12 strains) whereas in 2006 it was predominantly ST-1947 (7 out of 9 patient strains), a sequence type so far detected only in Finland. The strains belonging to the ST-22 complex comprised 13% in 1996 and 11% in 2006 of all patient strains in the particular peak seasons of domestic *Campylobacter* infections [20], suggesting that in these years certain specific source(s) may have existed. Additionally, we included in our study five strains, four chicken meat isolates from retail shops belonging to ST-22 from 1996 and one bovine isolate with ST-3892 (from 2003). Although ST-22 strains have also been isolated from animal sources (chicken, cattle, lamb and cows milk) in other countries, this particular ST seems to be rather uncommon (PubMLST). In fact, as found in our study, chicken meat could be a potential source of ST-22 strains as the samples from retail shops were from the same geographic area and the same time period as the patients strains. Even though there was a strong geographical association of ST-1947 with Finland [20], the potential source of this ST remained unknown and warrants further studies.

The study of the LOS locus classes combined with MLST data can provide epidemiologically valuable information since bacteria use different strategies in order to evade a host's immune system such as the ability to modify the structure of the surface-exposed macromolecules [27]. Limited data is available on the association of the LOS classes with ST complexes; however, studies on GBS associated strains have identified ST-22 strains to have LOS class A [8]. In the study of Mortensen et al. [24], sialylated LOS class A strains were significantly associated with reactive arthritis as well. Reactive arthritis has been a common postinfectious finding among Finnish campylobacteriosis patients. In the study of Schönberg-Norio et al. [11] on Finnish patients from 2002, musculoskeletal symptoms associated with *C. jejuni* enteritis were frequently reported by the patients (39%) and reactive arthritis was found in 4%. In an earlier Finnish study, reactive arthritis followed *Campylobacter* enteritis in 7% of patients [9]. Unfortunately, no data on the development of reactive arthritis was available for the patients from 1996 or 2006. Although LOS class A has been shown to be a significant neuropathogenic marker among *C. jejuni*, these types of strains have also been isolated from patients with uncomplicated enteritis [6], [24].

Comparative genomic microarray hybridization on *C. jejuni* strains with Penner serotype 19 (four strains have been MLST typed and belong to the ST-22 complex: GB28, GB18, GB2 and GB3) have shown that this group is homogeneous having a clonal structure regardless of disease outcome or geographical source of the strains [6]. Our study targeted certain bacterial properties, which may have a role in the host-bacterium interaction, showing that ST-22 complex strains have common characteristics and thus, extended the findings of Taboada et al. [6]. Previously, we found that all strains of this complex have the flagellar secreted protein *fspA1* gene which differed from the other FspA1 cluster strains by Thr→Ala substitution in the predicted protein sequence [28]. In addition, in the present study we detected by PCR [29] in all strains *Campylobacter* Invasion Antigen B (*ciaB*) and *cadF* which encodes a protein that interacts with the host extracellular matrix protein fibronectin (data not shown), these are both potential virulence determinants.

Despite the homogeneity observed and the fact that ST-22 and ST-1947 differ by only one nucleotide in the *glnA* locus, they had different PFGE genotypes and major phenotypic differences as well. Yabe and colleagues [19] who genotyped seven Japanese ST-22 complex strains (ST-22, ST-545 and ST-4051) by PFGE with *SmaI*, obtained similar profiles as ours for the ST-22 strains, revealing genotype similarity on distinct geographical regions. Our ST-22 strains were all GGT positive and could form biofilms, except one strain, while all those of ST-1947 were GGT negative and did not show any ability to form biofilms. In our study the difference in the GGT activity was the most remarkable biochemical difference between the strains. Moreover, the absence or presence of the gene has been performed in our previous study [30] and all strains that did harbour the *ggt* gene did express GGT activity. GGT is used in the glutamine and glutathione metabolism [30]. Although GGT is not expressed in all *C. jejuni* strains [30], [31], it was found among approximately 40% of the isolates [30] and is associated with certain clonal complexes and/or STs, e.g. ST-21 is GGT negative while ST-45 positive [32] and hosts [30]. Moreover, GGT has been shown in chicken model to be important in long lasting gut colonization, and *in vitro* it has been shown that GGT plays a significant role in *C. jejuni*-mediated apoptosis [31]. However the effective role of GGT as a virulence factor in human infections needs more studies. If the difference between ST-22 and ST-1947 has an impact on the disease outcome or host association in campylobacteriosis warrants further investigation.

Barnes et al. [31] showed that a *C. jejuni* Δ*ggt* mutant strain had higher motility when compared to the wild-type. In support of this, we observed that GGT negative strains had significantly higher motility than the strains which were GGT positive. In our study, GGT activity and motility on soft MH appeared negatively correlated. Mueller Hinton (MH) contains low amount of free L-Glutamine (∼0.2 mM) [33] and in order to investigate if substrates for GGT, L-glutamine (Gln) and Glutathione (GSH), as well as the end product L-glutamic acid (Glu) could have effect on the motility, we supplemented the medium with 20 mM of each compound. In our study the motility was not affected by the addition of Glu, but was increased in the media supplemented either with GSH or Gln in both ST-22 and ST-1947 strains. Nevertheless, the differences between the two groups were still statistically significant, suggesting that GGT activity was not the reason for motility differences on MH. In support of this, no effect on the motility of control strain *C. jejuni* 81-176 (GGT positive) was observed after the addition of Gln or GSH (data not shown). The explanation could be related to differing motility - or chemotaxis - associated genes among ST-22 and ST-1947, however, further studies are needed.An ability to form biofilm has been suggested to indicate a capability to survive outside the gut [34], to improve gut colonization [35] and to contribute to the stress tolerances during pathogenesis [35]. However no clear association of *in vitro* biofilm formation ability and virulence of *C. jejuni* has been shown to exist so far. One possible reason for the uncertainty of the role of biofilm on the pathogenesis of *C. jejuni* infections could be due to the absence of a standard method to measure this feature in this species. In this study, we found that several *C. jejuni* strains, including 81–176, showed differences in the strengths of pellicles when the assay was performed with Nutrient Broth or Muller-Hinton broth. Although strains that produced biofilm in Nutrient broth were also able to do it on Muller-Hinton, the visual score when using Muller-Hinton could induce to false-negatives, since the pellicle structure with this media is much weaker than with Nutrient Broth (data not shown). Our data pointed out the importance of media composition in the sensitivity on the biofilm determination on borosilicate tubes as previously described [36], suggesting Nutrient Broth to be a better candidate medium for this assay. As previously observed [34], strains with the same ST may present different phenotypes in biofilm formation. In our study, all the ST-22 strains formed *in vitro* biofilm, except one: strain FB5861. This strain had a slightly different PFGE pattern compared to the other ST-22 strains and had similar motility as ST-1947 strains. These results suggest that in the ST-22 different phenotypes may be associated with minor changes in the PFGE genotypes.

Several *in vitro* cell culture models for accessing both adhesion and invasion have been proposed using various cell lines, including HeLa, Int407 and Caco-2 cells [37]. However, it has been shown that Int407 and HeLa cells are more responsive to *C. jejuni* in terms of IL-8 secretion than Caco-2 cells [37]. Since Int407 cells have higher basal level of IL-8 than HeLa cells [37], we selected HeLa cells as a model to access the invasion and adhesion abilities as well as IL-8 stimulation by ST-22 complex strains. The adhesion and invasion capability of HeLa cells of our strains was widely distributed and no clear association with any other characteristics was seen. The four chicken isolates were not included for invasion, adhesion and stimulation of IL-8 tests, since we were finding that the interaction of other ST-22 strains was a strain-dependent characteristic. Invasion has been shown to be a common feature of *C. jejuni* strains studied *in vitro*, but no clear association with the severity of clinical symptoms has been found and it has been concluded to be a strain-dependent characteristic [38]. Although ST-22 strains presented significantly lower motility compared to ST-1947, the invasion and adhesion capabilities were similar and variation between strains was more pronounced than between the STs. Interestingly, Barnes et al. [31] observed that *C. jejuni Δggt* mutant strain which had higher motility than the parental strain, as mentioned above, also presented higher apparent invasion efficiency compared to the wild-type. Nevertheless, our data indicate that differences in motility appear to not affect the invasion and adhesion capability in HeLa cells. However, as discussed above, changes in the media or atmosphere composition can affect the motility of the strains on soft agar. Thus the assessment of motility and invasion *in vitro* cell cultures needs to consider the bacterial cultivation conditions. The strains of both major STs tended to induce the production of IL-8 at the same level. However, five strains of the ST-22 group promoted significantly lower production of IL-8 than the other strains, suggesting that also IL-8 induction could be a strain associated characteristic. Earlier, invasion has been shown to be necessary for high IL-8 production [38], however, we did not find any correlation between these two features.

In conclusion, our study aimed to characterize a collection of 22 clinical Finnish *C.jejuni* strains from the ST-22 complex, mostly isolated from human patients in either 1996 or 2006, and five animal strains. Although the ST-22 complex consisted of five separate STs, all strains expressed sialylated LOS locus class A_1_ and had similar adhesion and invasion capability. There were strong homogeny within each of the two major STs, ST-22 and ST-1947, with respect to all characteristics studied except for the parameters of the interaction with host cells. However, the two STs differed in many aspects such as PFGE genotypes, GGT production, motility and the ability to form *in vitro* biofilm. The results suggested that these two STs may have diverged from each other and acquired or lose different characteristics, possibly during host(s) colonization, forming their own clonal lineages within the ST-22 complex. Nevertheless, further studies including other strains belonging to ST-22 complex from different countries should be performed in order to verify this hypothesis.

## Materials and Methods

### Bacterial strains, growth conditions and DNA isolation

A total of 22 *C. jejuni* strains that were previously isolated from Finnish patients with domestically acquired infections in the Helsinki area between July to October in 1996 (12 strains) and 2006 (9 strains) were included [20], [26]. In addition, one human strain was isolated in summer 2003 [20], [26]. Three of the strains (FB6426, FB7273, FB7618) had Penner serotype 19 [39]. Moreover, four chicken meat isolates from 1996 with ST-22 and a bovine strain from 2003 with a Penner 19 and an ST-3892 [23], were included in this study. Detailed information regarding the strains is presented in Figure 1, while allelic differences are presented in Table 1.

**Table 1:** Multi Locus Sequence Typing housekeeping gene alleles for STs examined in this study from the ST-22 complex.

ST	*aspA*	*glnA*	*gltA*	*glyA*	*pgm*	*tkt*	*uncA*
22	1	3	6	4	3	3	3
1966	1	3	6	4	3	**276**	3
1947	1	**94**	6	4	3	3	3
3892	1	3	6	**3**	3	3	3
3996	1	**334**	6	4	3	3	3

Variable loci among ST-22 complex are underlined (alleles on the locus *glnA*, *glyA* and *tkt*).

Strains were taken from −70°C stock and cultivated on Nutrient Agar plus 5% of defibrinated horse blood (NBA, Oxoid) plates and incubated under microaerobic conditions (5% O_2_, 10% CO_2_ and 85% N_2_) at 37°C for 2 days. Care was taken to pass the strain no more than four times. DNA isolation was carried out as described previously [26].

### PFGE

The PFGE analysis was performed as described previously [4] using *Sma*I (New England Biolabs Inc.; 20U per sample) restriction enzyme. PFGE data were analyzed with BioNumerics V. 5.10 software (Applied Maths, Kortrijk, Belgium) using the Dice similarity coefficient, with 0.5% optimization and 1% tolerance. Clustering was done with the unweighted pair group method using arithmetic averages.

### LOS locus classes and SDS-PAGE profile

PCR reactions for screening the lipooligosaccharide (LOS) locus classes were performed using a set of 12 previously described primers (listed in Table 2) designed to identify the most common LOS classes [40]. PCRs were performed in a 25 µL volume; each reaction mixture contained 5 µL of DNA template and a PCR mixture consisting of 1x Optimized DyNAzyme buffer, 200 µM each deoxynucleoside triphosphate, 0.4 µM of each PCR primer, and 0.5 U/25 µL of DyNAzyme I DNA polymerase per reaction (final concentrations). The cycling conditions were as follows: initial denaturation step of 3 minutes at 94°C; 30 cycles of 25 seconds at 94°C, 25 seconds at 52°C and 1 minute at 72°C; and a final extension for 5 min at 72°C.

**Table 2:** Primers from Parker et al. (2005) used in this study and assignment of lipooligosaccharide locus classes based upon PCR results.

LOS locus class ORF	A1	A2	B1	B2	C	D	E	F	H	Primer 1 5′ →3′	Primer 2 5′ →3′
12	+	+	+	+	+	+	+	+	+	GCCACAACTTTCTATCATAATCCCGC	CGCCGTAACTCAAACGCTCATCTATT
6ab1	+	-	+	-	-	-	-	-	-	CAAGGGCAATAGAAAGCTGTATCA	ACAAGCACTTCATTCTTAGTATTACAAAT
6ab2	-	+	-	+	-	-	-	-	-	TCATCTTGCCAACTTATAATGTGGA	TCTAGCGATATTAAACCAACAGCCT
7ab	+	+	+	+	-	-	-	-	-	ACTACACTTTAAAACATTTAATCCAAAATCA	CCATAAGCCTCACTAGAAGGTATGAGTATA
5bII	-	-	+	+	-	-	-	-	-	CTGTGATGATGGGAGTGAAGAGC	GGTAATCGTTTCGGCGGTATT
6c	-	-	-	-	+	-	-	-	-	GTAGTAGATGATTGTGGTAATGATAAA	ATAGAATTGCTATTTACATGCTGG
17d	-	-	-	-	-	+	-	-	-	TTGAACAACCTGCTTATGAGCTTTAT	TTTCTTTAGTGAATCTTCCCACGC
18df	-	-	-	-	-	+	-	+	-	GCAGCAAGAAATAATGGTGTTAAAC	AAATAATCATCC AAACATTCCTGAA
19df	-	-	-	-	-	+	-	+	-	AAAATTTCCGTCATAATCC CAATCT	TATCAGGTAAATCTTGAATGATAAAGTCA
20df	-	-	-	-	-	+	-	+	-	GTCTTTTAAGAGCTAGATATGAAGGAG	ATTAATGCATCTTCTGCCATAATTA
26e	-	-	-	-	-	-	+	-	-	ATATTGCCGTTAATTCATTACAGTT	TTTGAGCGATAATTTTAAATCCATC
27e	-	-	-	-	-	-	+	-	+	GTAGATGATTGTTCAAATGATAATAGCACA	GTTTTCAGATTCTAAGGCCATTATTCC

*C. jejuni* LOS profiles and sialylation of LOS were assessed using silver stained SDS-PAGE gel. Briefly, strains were harvested in sterile water from overnight growth on NBA plates to an OD_600_ of 0.200 and treated with proteinase K (1 mg/mL) for 1.5 hours at 56°C. After the incubation, samples were boiled for 10 minutes and subsequently divided into two tubes, one used as a control (with water) and the other treated with neuraminidase (0.4 U/mL) from *Clostridium perfringens* (Sigma), and incubated overnight at 37°C. Electrophoresis was performed on precasted polyacrylamide 15% Tris-HCl gel (BioRad) followed by silver-staining.

### Adhesion, Invasion and IL-8 production

HeLa cells (ATCC/CCL-2) were grown and maintained in Dulbecco's modified Eagle's growth medium (DMEM, Invitrogen) supplemented with 10% of Foetal Bovine Serum (FBS), 100 U/mL of penicillin and 0.1 mg/mL of streptomycin (Pen Strep, Gibco, Invitrogen), with incubation conditions at 37°C with 5% CO_2_. For all tests cells were seeded in 24-well tissue-culture plates (Nunc) at 5×10^5^ cells/mL final concentration in fresh medium without antibiotics and incubated overnight. The adhesion and invasion of *C. jejuni* bacteria was assessed by the gentamicin protection assay as described previously [41], using a Multiplicity of Infection (MOI) of 100. In brief, bacterial suspensions were added to the cells and incubated for two hours. For invasion determination, 200 µg gentamicin/mL (Invitrogen) per well were added and the plate was incubated for a further 1.5 h. Monolayers were washed and lysed with 0.2% (v/v) Triton X-100. The CFU/ml were determined on NBA. In order to estimate adherence, the same procedure was performed, however instead of gentamicin, DMEM was added. The CFU/mL of adherent cells was determined by the difference of the CFU/mL of plain culture and CFU/mL of gentamicin-treated culture. The levels of adherence and invasion are presented as log CFU/mL.

In order to measure the IL-8 secretion, HeLa cells were infected with *C. jejuni* strains for 24 h, cell supernatants were collected into Eppendorf tubes, particulate material was removed by centrifugation and the samples aliquoted and stored at −70°C until further analysis. Quantification of IL-8 secretion into the cell growth medium from infected and uninfected cells was performed by Enzyme Linked Immunosorbent Assay (ELISA) using the Quantikine Human CXCL8/IL-8 kit (R&D Systems, inc., Minneapolis, USA), according to the manufacturer's instructions.

### *In vitro* phenotypic studies

All phenotypic assays were carried out in duplicate and experiments were performed three times for verification. Biofilm formation was evaluated as previously described [35] with minor modifications. Briefly, after overnight incubation, bacteria were collected from the NBA plates and suspended in PBS to an OD_600_ of 0.200, diluted 100 times in Nutrient Broth n°2 (Oxoid) and the suspension was put into Duran borosilicate glass tubes. The tubes were incubated under microaerobic conditions at 37°C without agitation for 48 h. The broth (planktonic) was carefully removed; the tubes were stained with 1% crystal violet in ethanol, rinsed with distilled water and dried. The strength of the biofilms were assessed visually and scored as positive or negative to form biofilm at the interface between between air-liquid (pellicle).

For the determination of Y-glutamyl transpeptidase activity (GGT), bacteria were suspended in PBS and OD_600_ adjusted to 0.800. After centrifugation the supernatant was removed. 400 µL reaction buffer was added as described previously [42] and the bacterial cells were incubated at 37°C for 30 minutes. Reactions were stopped by the addition of 40 µL 20% acetic acid. Simultaneously, bacterial suspensions were boiled and tested as negative controls. *p*-nitroaniline liberated from the substrate analog L-Y-glutamyl-p-nitroanilide was measured at 405 nm with Multiskan Ascent Microplate Reader (Thermo Electron Corporation) and the strains were scored as GGT positive or negative.

Motility was determined using semisolid motility agar consisting of MH broth (Difco) supplemented with 0.4% agar. The plates were inoculated using 1 µL of bacteria in MH broth of a suspension with OD_600_ of 0.250 diluted 10 times. The plates were incubated at 37°C in a microaerobic atmosphere, with supplementation of hydrogen (jar with gas composition of CO_2_ 10%; H_2_ 5%; N_2_ 85%) or without supplementation of hydrogen (microaerobic incubator ThermoForma [Thermo Electron Corporation, Marietta, OH], with a gas composition of CO_2_ 10%; O_2_ 5%; N_2_ 85%), and the diameter of the area of motility (mm) was measured after 24 h and 48 h. Additional motility studies were performed by adding to the soft MH agar 20 mM of L-Glutamine (Gln), Gluthatione reduced (GSH) or L-Glutamic acid (Glu) (Sigma), in a microaerobic atmosphere without supplementation of hydrogen for 24 h. The concentration used was the same as the one indicated by Guccione et al. [33].

### Statistical analysis

Statistical analysis was performed using analysis of variance, One-way ANOVA followed by Bonferroni post-test with a cut-off of 0.05 and for motility comparison between ST-22 and ST-1947 strains, unpaired two-tailed *t*-test was carried out. Error bars in the graphs in all figures were calculated as Standard Error of the Mean (SEM). The analyses were performed using GraphPad Prism version 4.03 for Windows, GraphPad Software, San Diego California USA, www.graphpad.com.

## Supporting Information

Table S1 **Motility of the different Sequence Types within the ST-22 complex.** The motility of a total 27 C. jejuni strains was evaluated on soft agar (Muller-Hinton supplemented with 0.4% of agar) at 37°C after 24 h and 48 h, incubated microaerobically with or without hydrogen enrichment. In general, strains exhibited higher motility when incubated microaerobically with hydrogen. Data are presented as mean ± SEM of duplicate.
(DOC)
