# Supplementary material for: Finnish Campylobacter jejuni Strains of Multilocus Sequence Type ST-22 Complex Have Two Lineages with Different Characteristics
Source: PLoS One. 2011 Oct 24;6(10):e26880. doi: 10.1371/journal.pone.0026880 (PMC3200363; doi:10.1371/journal.pone.0026880)
Supplement: Table S1 — Motility of the different Sequence Types within the ST-22 complex. The motility of a total 27 C. jejuni strains was evaluated on soft agar (Muller-Hinton supplemented with 0.4% of agar) at 37°C after 24 h and 48 h, incubated microaerobically with or without hydrogen enrichment. In general, strains exhibited higher motility when incubated microaerobically with hydrogen. Data are presented as mean ± SEM of duplicate. (DOC) [file pone.0026880.s001.doc]

**Table S1:** Motility of the different Sequence Types within the ST-22 complex. The motility of a total 27 *C. jejuni* strains was evaluated on soft agar (Muller-Hinton supplemented with 0.4% of agar) at 37°C after 24h and 48h, incubated microaerobically with or without hydrogen enrichment. In general, strains exhibited higher motility when incubated microaerobically with hydrogen. Data are presented as mean ± SEM of duplicate.

| **Strain** | **ST** | Microaerobic atmosphere without supplementation of hydrogen | | Microaerobic atmosphere with supplementation of hydrogen | |
| --- | --- | --- | --- | --- | --- |
|  |  | 24h | 48h | 24h | 48h |
| FB5861 | 22 | 9.5±1.50 | 41.0±2.52 | 23.5±2.50 | 46.7±2.85 |
| FB6170 | 22 | 4.7±0.67 | 33.3±1.89 | 15.5±2.50 | 41.0±2.35 |
| FB6329 | 22 | 5.3±0.33 | 30.0±2.86 | 16.5±3.50 | 42.0±2.04 |
| FB6426 | 22 | 5.3±0.33 | 20.7±2.33 | 12.0±4.00 | 34.8±0.95 |
| FB7143 | 22 | 5.3±0.33 | 27.8±1.83 | 12.5±3.50 | 38.7±2.73 |
| FB7273 | 22 | 5.7±0.67 | 26.5±3.42 | 14.0±2.00 | 37.3±2.32 |
| FB7373 | 22 | 5.7±1.20 | 24.0±3.24 | 14.5±2.50 | 38.3±1.93 |
| FB7454 | 22 | 6.3±0.88 | 30.5±2.72 | 15.0±1.00 | 40.8±2.84 |
| FB7511 | 22 | 6.0±1.00 | 22.3±1.86 | 15.0±1.00 | 39.5±3.30 |
| FB7618 | 22 | 5.0±1.16 | 29.0±3.22 | 12.5±1.50 | 34.0±2.86 |
| 76577 | 22 | 6.7±0.33 | 31.8±1.89 | 14.0±4.00 | 37.5±2.78 |
| 22A | 22 | 6.0±0.00 | 32.0±1.00 | 15.5±1.50 | 42.0±2.00 |
| 29A | 22 | 5.0±1.00 | 28.5±1.50 | 12.5±2.50 | 37.0±1.00 |
| 30A | 22 | 5.5±0.50 | 32.5±1.50 | 14.0±2.00 | 40.0±0.00 |
| 38B | 22 | 4.5±0.50 | 32.0±0.00 | 14.0±2.00 | 39.5±0.50 |
| FB7095 | 1947 | 11.5±3.50 | 37.7±2.73 | 20.5±7.50 | 54.3±6.84 |
| FB7437 | 1947 | 13.5±1.50 | 41.0±3.22 | 20.5±6.50 | 49.0±2.65 |
| 76430 | 1947 | 12.0±3.00 | 39.0±4.00 | 17.5±6.50 | 48.7±1.76 |
| 76433 | 1947 | 8.5±2.50 | 39.0±3.51 | 15.5±7.50 | 49.0±4.40 |
| 76443 | 1947 | 10.5±3.50 | 41.3±2.73 | 16.0±7.00 | 50.7±7.54 |
| 76451 | 1947 | 10.5±2.50 | 37.0±3.61 | 16.5±5.80 | 51.0±5.20 |
| 76455 | 1947 | 12.0±4.00 | 42.3±3.48 | 15.5±6.50 | 45.0±1.73 |
| 76458 | 1947 | 12.5±5.50 | 38.7±5.36 | 19.5±5.50 | 48.7±3.48 |
| 76468 | 1947 | 12.5±5.50 | 38.3±5.89 | 18.0±5.00 | 49.7±7.84 |
| 73715 | 1966 | 10.0±0.00 | 37.0±1.53 | 16.5±1.50 | 46.7±0.88 |
| 3673-1 | 3892 | 5.5±1.50 | 16.3±0.88 | 6.5±0.50 | 32.3±4.37 |
| 76781 | 3996 | 10.5±1.50 | 40.5±1.50 | 19.0±3.00 | 53.5±0.50 |
